# Both Transmembrane Domains of BK β1 Subunits Are Essential to Confer the Normal Phenotype of β1-Containing BK Channels

**DOI:** 10.1371/journal.pone.0109306

**Published:** 2014-10-02

**Authors:** Guruprasad Kuntamallappanavar, Ligia Toro, Alex M. Dopico

**Affiliations:** 1 Department of Pharmacology, College of Medicine, The University of Tennessee Health Science Center, Memphis, Tennessee, United States of America; 2 Departments of Anesthesiology and of Molecular and Medical Pharmacology, University of California Los Angeles, David Geffen School of Medicine, Los Angeles, California, United States of America; University of Houston, United States of America

## Abstract

Voltage/Ca^2+^_i_-gated, large conductance K^+^ (BK) channels result from tetrameric association of α (slo1) subunits. In most tissues, BK protein complexes include regulatory β subunits that contain two transmembrane domains (TM1, TM2), an extracellular loop, and two short intracellular termini. Four BK β types have been identified, each presenting a rather selective tissue-specific expression profile. Thus, BK β modifies current phenotype to suit physiology in a tissue-specific manner. The smooth muscle-abundant BK β1 drastically increases the channel's apparent Ca^2+^_i_ sensitivity. The resulting phenotype is critical for BK channel activity to increase in response to Ca^2+^ levels reached near the channel during depolarization-induced Ca^2+^ influx and myocyte contraction. The eventual BK channel activation generates outward K^+^ currents that drive the membrane potential in the negative direction and eventually counteract depolarization-induced Ca^2+^ influx. The BK β1 regions responsible for the characteristic phenotype of β1-containing BK channels remain to be identified. We used patch-clamp electrophysiology on channels resulting from the combination of smooth muscle slo1 (cbv1) subunits with smooth muscle-abundant β1, neuron-abundant β4, or chimeras constructed by swapping β1 and β4 regions, and determined the contribution of specific β1 regions to the BK phenotype. At Ca^2+^ levels found near the channel during myocyte contraction (10 µM), channel complexes that included chimeras having both TMs from β1 and the remaining regions (“background”) from β4 showed a phenotype (V_half_, τ_act_, τ_deact_) identical to that of complexes containing *wt* β1. This phenotype could not be evoked by complexes that included chimeras combining either β1 TM1 or β1 TM2 with a β4 background. Likewise, β “halves” (each including β1 TM1 or β1 TM2) resulting from interrupting the continuity of the EC loop failed to render the normal phenotype, indicating that physical connection between β1 TMs *via* the EC loop is also necessary for proper channel function.

## Introduction

**Figure 1:**
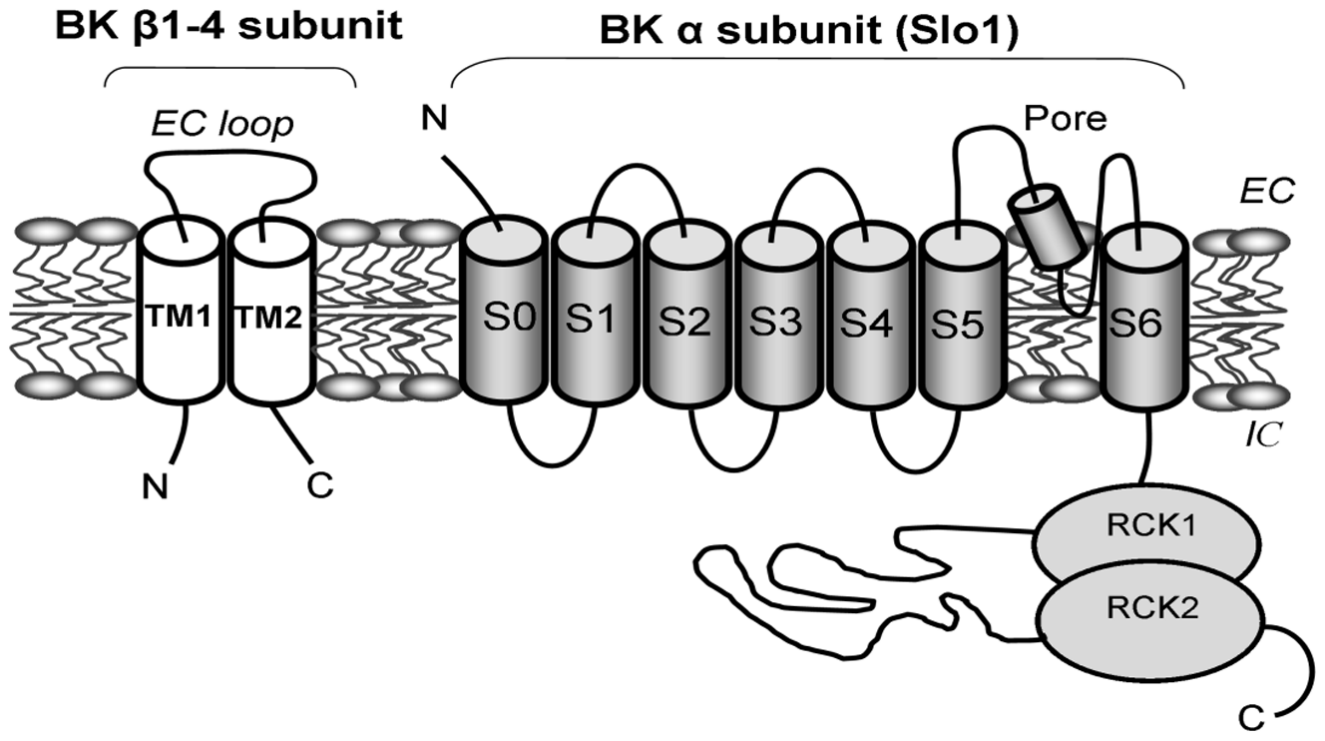
Schematic structure of β1 subunit-containing BK channel. Cartoon showing a slo1-β1 subunit heterodimer. The channel-forming slo1 subunit includes transmembrane (TM) segments S0-S6 and intracellular Regulatory of Conductance for K^+^ (RCK) domains, these domains including distinct residues that participate in sensing changes in Ca^2+^_i_. Four slo1 monomers assemble to render fully functional BK channels. All four types of β subunits identified so far contain a similar design that includes intracellular N- and C-terminals, two transmembrane domains (TM1 and TM2), and an EC loop. *EC* and *IC* correspond to the extracellular and intracellular sides of the membrane.

Large conductance, voltage- and Ca^2+^-gated K^+^ (BK) channels are ubiquitously expressed and thus, control numerous physiological processes [1]–[3]. Functional BK channels result from tetrameric association of channel-forming proteins termed α (slo1) subunits. These subunits contain a transmembrane S1-S6 region that is primarily responsible for ion permeation and voltage-gating, and conserved in all members of the TM6 superfamily of voltage-gated K^+^ (K_V_) channels. In addition, slo1 proteins distinctively contain: 1) a transmembrane (TM) segment S0 that leads to an extracellular N-end [4], which participates in voltage-sensing [5]–[7], and 2) a large cytosolic C-end (CTD), which allows the BK channel to increase activity in response to increased Ca^2+^_i_ within the physiological range [8] (**Fig. 1**).

In most tissues, however, slo1 is associated with accessory proteins termed β subunits. Several BK β subunit-coding sequences have been cloned (β1-β4), all their protein products sharing a common design: short intracellular (IC) N- and C-ends and two TMs (TM1, TM2) connected by an extracellular (EC) loop (**Fig. 1**). Remarkably, BK β subunit type expression is highly tissue-specific, and the modification in slo1 current introduced by a given β type helps to define channel phenotypes that suit cell physiology in a tissue-specific manner [2], [9]. BK β1 subunit abundant expression in smooth muscle (SM) results in a robust increase in the native channel's apparent Ca^2+^_i_ sensitivity. Therefore, SM BK channels drastically increase activity when slo1 sensors are exposed to changes in Ca^2+^_i_ from the sub-µM levels found under resting conditions to ∼4-30 µM, these levels being reached in the vicinity of the BK channel's Ca^2+^ sensors in the contracting SM cell [10]. The resulting hyperpolarizing outward K^+^ currents generated by β1–containing BK channels negatively feed-back on Ca^2+^_i_ increase and thus, limit SM contraction [11].

The increase in channel's apparent Ca^2+^_i_ sensitivity induced by BK β1results from complex regulation of slo1 gating by this regulatory subunit, including modulation of Ca^2+^_i_-channel protein interaction itself and voltage-sensor activation [1], [12], [13], and reduction in voltage-dependence steepness [14]–[16]. BK β1 subunits, however, decrease channel activity at sub-µM Ca^2+^_i_ by reducing intrinsic gating (i.e., the capability of the channel to gate in absence of voltage-activation, Ca^2+^_i_-binding or any other regulator) [1], [13]. BK β1 also slows activation and deactivation kinetics [15], [17] and participates in channel sensitivity to 17β-estradiol [18] and cholane steroids an non-steroidal analogs [19], [20]. While the changes in BK channel phenotype introduced by β1 have been studied in detail, identification of the specific BK β1 regions that participate in determining the characteristic β1-containing BK channel phenotype remains unresolved.

Data from mslo1/dslo chimeras seem to indicate that the region expanding from the N-end to the S0-S1 loop contributes to modulation of apparent Ca^2+^_i_ sensitivity by β1 or β2 subnits [21]. Cysteine disulfide cross-linking studies attribute to the EC sides of β2, β3, and β4 TM1 and TM2 topological associations with slo1 that are similar to those of β1 EC sides [22], [23]. In spite of these proposed topological similarities, ion channel current phenotypes resulting from heteromeric association between slo1 and each β type differ markedly [reviewed in 9]. Moreover, neither cysteine substitutions *per se* nor disulfide cross-linking in EC regions have major effects on several key parameters of BK ionic current phenotype such as current half-voltage activation (V_half_), activation or deactivation kinetics [5], strongly suggesting that regions non-accessible to cysteine substitutions (e.g., TMs) could play a key role in determining the phenotype of β1-containing BK channels. Consistent with this possibility, β1 EC loop Ala substitutions that altered some gating parameters failed to eliminate the characteristic leftward-shift along the voltage axis introduced by β1 [24]. On the other hand, functional studies from BK channels made of slo1 and chimeric β1/β2 subunits indicate that β C- and N-ends play a significant role in determining the channel phenotype, yet a shared modulatory role by TMs has been hypothesized [16].

To determine whether BK β1 TM1, TM2 or both are critical to provide the characteristic ion current phenotype of BK β1-containing BK channels, we combined surface protein biotinylation assays with patch-clamp studies under wide voltage and Ca^2+^_i_ ranges (which included the values found in the SM myocyte under physiological conditions) on heteromeric BK complexes resulting from the association of rat cerebral artery SM slo1 (“cbv1”) with engineered BK β1. Using this approach, we demonstrate that: 1) neither TM is sufficient but both are necessary to establish the characteristic phenotype of BK β1-containing BK channels, and 2) physical connection between both TMs *via* the EC loop is necessary to maintain such phenotype. This information is important to begin to understand the unique role of BK β1 in regulating channel function and cell physiology, and for future rationale design of ligands that selectively target β1-containing BK channels.

## Experimental Procedures

### Ethics statement

Care of animals and experimental protocols (internal protocol #1078) were reviewed and approved by the Institutional Animal Care and Use Committee at the University of Tennessee Hlth. Sci. Ctr., which is an Association for Assessment and Accreditation of Laboratory Animal Care-accredited institution (A3325-01; 07/10/2012-07/31/2016).

### cRNA preparation and injection into *Xenopus laevis* oocytes

Cloning, expression and functional characterization of cbv1 (AY330293) are provided elsewhere [25], [26]. BK hβ1, hβ4, and hβ1/hβ4 chimeric cDNAs (β1TMs_4_, β4TMs_1_, β4TM1_1_ and β4TM2_1_) were cloned in Dr. Ligia Toro's lab (UCLA). In addition, we engineered two “split” chimeras from the β4TMs_1_ to render: 1) “N-half chimera”, which contained the N-terminus from β4, TM1 from β1 and proximal half of β4 EC loop; 2) “C-half chimera”, which contained the distal half of β4 EC loop, TM2 from β1, and the C-terminus from β4. Flag tag was inserted at N-terminus of C-half chimera to detect during surface biotinylation. All constructs were verified by automated sequencing (Molecular Resource Center, University of Tennessee Health Science Center). These cDNAs were subcloned into pOx for oocyte expression.

cRNA was dissolved in diethyl polycarbonate-treated water at 10 (cbv1) and 30 (β1) ng/µl; 1 µl aliquots were stored at −70°C. Oocytes were removed from *Xenopus laevis* (Xenopus Express), prepared and cRNA-injected as described [27]. The interval between injection and patch-clamping was ≥36 h.

### Electrophysiology

Oocytes were prepared for patch-clamp electrophysiology as previously described [27], with Inside out (I/O) patches being used to record macroscopic ion current. Bath and electrode solutions contained (mM): 130 K-gluconate, 5 EGTA, 1.6 HEDTA, 15 HEPES; pH 7.4. Variant amounts of CaCl_2_ and MgCl_2_ were used to set the free Ca^2+^ at the desired level and free Mg^2+^ to 1 mM. Free Ca^2+^ and Mg^2+^ were calculated using Max Chelator (C. Patton; Stanford). Actual Ca^2+^ levels in solution were determined experimentally with Ca^2+^-sensitive electrodes (Corning) [27]. For experiments in nominal zero Ca^2+^_i_, EDTA was substituted by 5 mM EGTA and no Ca^2+^ was added to the recording solutions. Free [Ca^2+^] in this nominal zero Ca^2+^_i_ solution is 0.5 nM [21]. In the experiments where the free Ca^2+^ was set to <1 µM, 1.6 mM HEDTA was omitted from the solution. All chemicals were purchased from Sigma.

Patch electrodes were pulled from glass capillaries (Drummond). The procedure gave tip resistances of 3–5 MΩ when filled with electrode solution. Experiments were carried out at room temperature (21°C). BK currents were acquired using an EPC8 (HEKA Electronics) amplifier and digitized using Digidata 1320A-pCLAMP8 (Molecular Devices). Macroscopic currents were evoked from a holding potential of −80 mV by 100 ms-long, 10 mV depolarizing steps from −150 to +150–200 mV. Standard P/4 leak subtraction routine was applied using a built-in function in pCLAMP. Currents were low-pass filtered at 1 kHz and sampled at 5 kHz.

Conductance-Voltage (G-V) relations were determined from the tail current amplitude, as described [15]. Resulting G/G_max_-V plots were fitted to a Boltzmann function of the type *G*(*V*) = G_max_/1+exp[(−*V*+*V_1/2_*)/*k*]. Boltzmann fitting routines were run using the Levenberg-Marquardt algorithm to perform nonlinear least squares fits. Macroscopic current activation and deactivation data were fitted to standard exponential functions using a Chebyshev approximation. Time constant for current activation (τ_act_) was measured at the voltage at which the channel reached maximal steady-state activity (V_max_) while deactivation time constant (τ_deact_) was measured after voltage reached V_max_ and then stepped down to −80 mV [17]. Data fitting and plotting were performed using Clampfit 9.2 (Molecular Devices) and Origin 8.5 (OriginLab).

### Detection of N-half and C-half chimeric proteins on the cell membrane surface by biotinylation

Presence of N-half and C-half chimeric proteins on the membrane surface of *Xenopus laevis* oocytes was detected using the Pierce Cell Surface Protein Isolation kit (Thermo Scientific) following the manufacturer's instructions. Immediately prior to the biotinylation-based labeling and separation of membrane surface proteins, the oocyte's follicular layer was removed to allow access of kit reagents to the cell membrane. The purified surface protein fraction was analyzed by Western blotting.

### Western blotting

Purified surface protein fraction for biotinylation (30 µg/lane) was separated on a 4–15% SDS-polyacrylamide gel and transferred onto PVDF (polyvinylidene difluoride) membranes. The membranes were then blocked with 5% non-fat dry milk made in tris-buffered saline containing 0.1% Tween 20 for 2 hrs. Membranes were then incubated with appropriate primary antibodies overnight at 4°C in Tris-buffered saline (TBS) with 0.1% Tween 20 (TBS-T) and 5% nonfat dry milk. Membranes were then incubated with appropriate horseradish peroxidase-conjugated secondary antibodies (1∶10,000 dilution; Milipore) for 1–2 hrs at room temperature. Proteins were then visualized using SuperSignal West Pico Chemiluminescent Substrate kit (Thermo Fisher Scientific). A slo1β4 antibody (1∶200 dilution; Alamone) was used to recognize the N-terminus of β4 subunit, and a mouse monoclonal anti-FLAG M2 antibody (1∶200 dilution; Sigma Aldrich) was used to detect the C-half chimera.

### Statistics

Analysis was performed using InStat 3.0 (GraphPad). Data were analyzed with one-way ANOVA followed by Tukey's multiple comparison test [28]. Significance was set at P<0.05. Data are expressed as mean±SEM; n = number of patches, each patch obtained from a separate oocyte.

## Results

### β1 TMs regulate BK current phenotype

**Figure 2:**
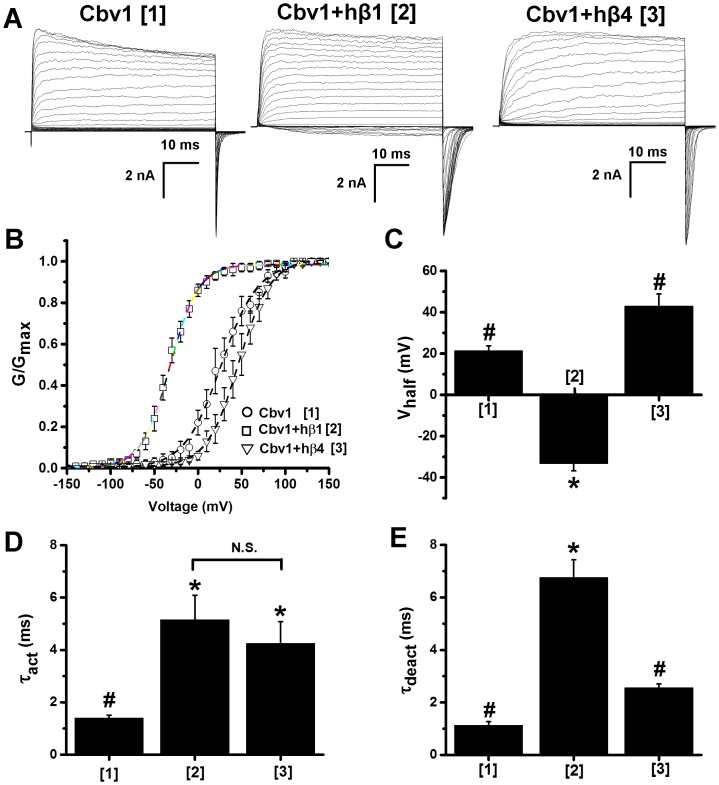
Characteristic BK current phenotype of channels made of cbv1± β1 or β4 subunits. Representative traces of macroscopic current recordings and averaged G/G_max_-V plots (B) obtained from I/O oocyte membrane patches expressing cbv1 (construct 1), cbv1+hβ1 (construct 2) or cbv1+hβ4 (construct 3); Ca^2+^_i_ = 10 µM. Bar graphs show averaged V_half_ (C), activation (D) and deactivation (E) time constants (τ_act_, τ_deact_, respectively) obtained for cbv1, cbv1+hβ1, and cbv1+hβ4; Ca^2+^_i_ = 10 µM. *Different from cbv1 (P<0.05); ^#^Different from cbv1+β1 (P<0.05). Error bars correspond to SEM; each point represents the average of ≥4 patches.

When considering all BK β subunit types, primary alignment of β1 vs. β4 reveals the highest number (56%) of non-identical and non-conserved residues [17]. Moreover, β1 vs. β4 co-expression with slo1 proteins renders BK channels with a different phenotype: noteworthy, β1 subunits greatly increase the apparent Ca^2+^ sensitivity of the channel at Ca^2+^_i_ concentrations>1 µM whereas the β4 subunit effect is rather limited, requiring ≥30 µM Ca^2+^_i_ to be observed [1], [15], [17]. Thus, we used chimeras resulting from swapping hβ1/hβ4 TM regions (whether individually or two at a time) to determine the contribution of β1 TMs to the β1-containing BK channel phenotype. These β1/β4 chimeras have been routinely used in our laboratory, their surface expression and function being confirmed by pharmacological profiling as described elsewhere [29]. Macroscopic ionic BK currents mediated by cbv1±hβ (*wt* β1, *wt* β4 or β1/β4 chimeras) were evoked by depolarizing steps (Materials and Methods) from inside-out (I/O) macropatches exposed to a wide Ca^2+^_i_ range (nominal zero-100 µM). This range includes the Ca^2+^_i_ faced by BK Ca^2+^-sensors that is required for cerebrovascular SM BK channels to fulfill their physiological role, that is, negatively feedback on depolarization-induced Ca^2+^ entry and SM contractility [4–30 µM; 10]. To identify the ion channel phenotype of the resulting BK channel complexes, we obtained G/G_max_-V plots, τ_act_ and τ_deact_ from each macropatch (ionic current traces shown in **Fig. 2A**). The time constants τ_act_ and τ_deact_ are widely recognized as indicators of BK current kinetics while G/G_max_-V plots were obtained to extrapolate V_half_ This parameter is indicator of overall BK channel activity, including both Ca^2+^_i_-dependent and Ca^2+^_i_-independent gating components [15], [30].

As shown for other slo1 channels [16], [17], [31], co-expression of hβ1 with cbv1 markedly left-shifted the G/G_max_-V plot along the voltage axis, leading to a ∼50 mV decrease in V_half_ at physiological, 10 µM Ca^2+^_i_: V_half_ = 21.42±2.39 and −33.22±3.56 mV for cbv1 and cbv1+hβ1, respectively (P<0.05) (**Figs. 2B,C** **and S1A**). Consistent with previous studies [16], [17], [31], the hβ1-driven shift in V_half_ increased as Ca^2+^_i_ was raised above ∼1 µM (**Fig. S1A**). Results underscore that µM Ca^2+^_i_ levels, while not necessary (**Figs. 2BC and** **S1A**), are optimal for β1-modulation of slo1, this modulation resulting in increased apparent Ca^2+^ sensitivity and thus enhanced steady-state current [12], [14], [15], [32]. In addition to its effect on cbv1 current V_half_, hβ1 remarkably increased τ_act_ and τ_deact_: at 10 µM Ca^2+^_i_, τ_act_ and τ_deact_ changed from 1.40±0.10 and 1.13±0.13 ms to 5.16±0.93 and 6.76±0.67 ms, for cbv1 and cbv1+hβ1, respectively (P<0.05 for both constants) (**Fig. 2D,E**). These changes are also in agreement with data from β1±slo1 other than cbv1 documenting a slowing down of macroscopic current activation and deactivation kinetics by BK β1 subunits [12], [15], [17].

In contrast to hβ1, hβ4 expression increased cbv1 V_half_ at 0.3–10 µM Ca^2+^_i_ while mildly decreasing V_half_ at 30–100 µM Ca^2+^_i_ (**Fig. 2B,C** **and S1A**). At 10 µM Ca^2+^_i_, hβ4 markedly increased τ_act_: 1.40±0.1 ms in cbv1 *vs*. 4.25±0.83 ms in cbv1+hβ4 (P<0.05), and exerted a mild effect on τ_deact_ (**Fig. 2D,E**). Collectively, the β4-introduced changes in V_half_, τ_act_ and τ_deact_ over cbv1 values are similar to those reported with β4 and other slo1s [9], [17].

**Figure 3:**
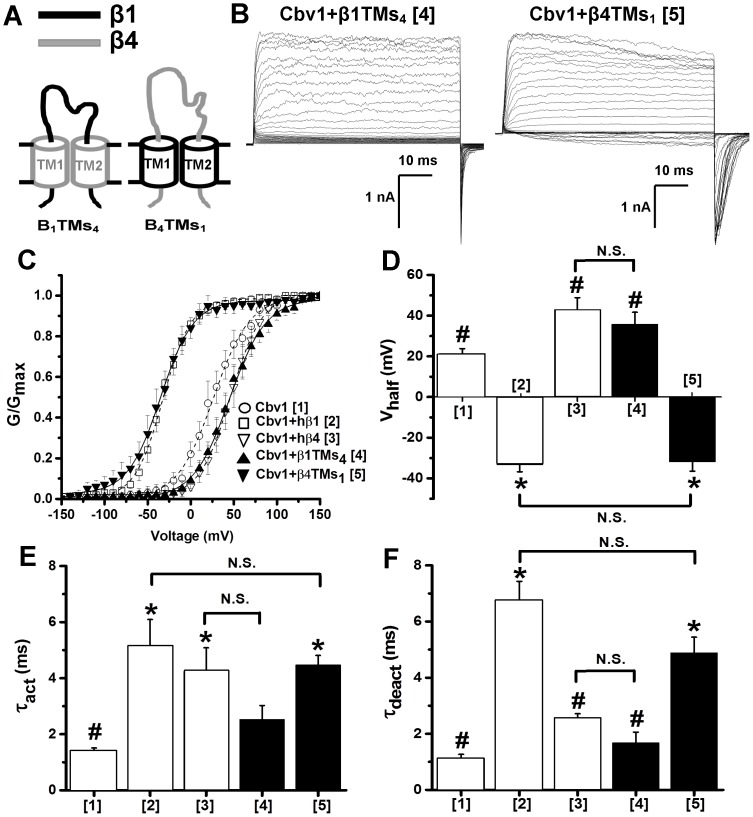
Both TMs of β1 are required for conferring the characteristic phenotype of β1-containing BK channel complexes. (A) Cartoons depicting the chimeric constructs obtained by swapping TM protein regions between hβ1 and hβ4 subunits. Regions from β1 and β4 are given in black and grey, respectively. (B) Macroscopic current recordings obtained from I/O oocyte membrane patches expressing cbv1+β1TMs_4_ (construct 4) and cbv1+β4TMs_1_ (construct 5) Ca^2+^_i_ = 10 µM. (C) Averaged G/G_max_-V plots of constructs 1–5 obtained at Ca^2+^_i_ = 10 µM. Averaged V_half_ (D), activation (E) and deactivation (F) time constants (τ_act_, τ_deact_, respectively) from constructs 1–5 obtained at 10 M Ca^2+^_i_. *Different from cbv1 (P<0.05); ^#^Different from cbv1+β1 (P<0.05). Error bars correspond to SEM; each point represents the average of ≥4 patches.

We next co-expressed cbv1 with chimeric β1TMs_4_ subunits that contained hβ4 TMs on hβ1 “background” (i.e., hβ1 EC loop and IC ends; **Fig. 3A**). Cbv1 channels co-expressed with these chimeras displayed V_half_-Ca^2+^_i_ plots, τ_act_ and τ_deact_ that matched those of cbv1+hβ4 (P>0.05) while differing from those of cbv1+hβ1 (**Figs. 3C-F** **and S1B**). Conversely, cbv1+chimeric β4TMs_1_ that contained hβ1 TMs introduced to an hβ4 background (**Fig. 3A**),), showed a phenotype that matched that of cbv1+hβ1: V_half_-Ca^2+^_i_ plot across all Ca^2+^_i_ tested (0.3–100 µM; **Fig 3B-D** **and S1B**), τ_act_ (**Fig 3E**) and τ_deact_ (**Fig 3F**) were all similar to those from cbv1+hβ1 (P>0.05). These results indicate that β1 TMs (but not the β1 background) are critical to support the major characteristics of steady-state ionic current generated by β1-containing BK protein complexes.

**Figure 4:**
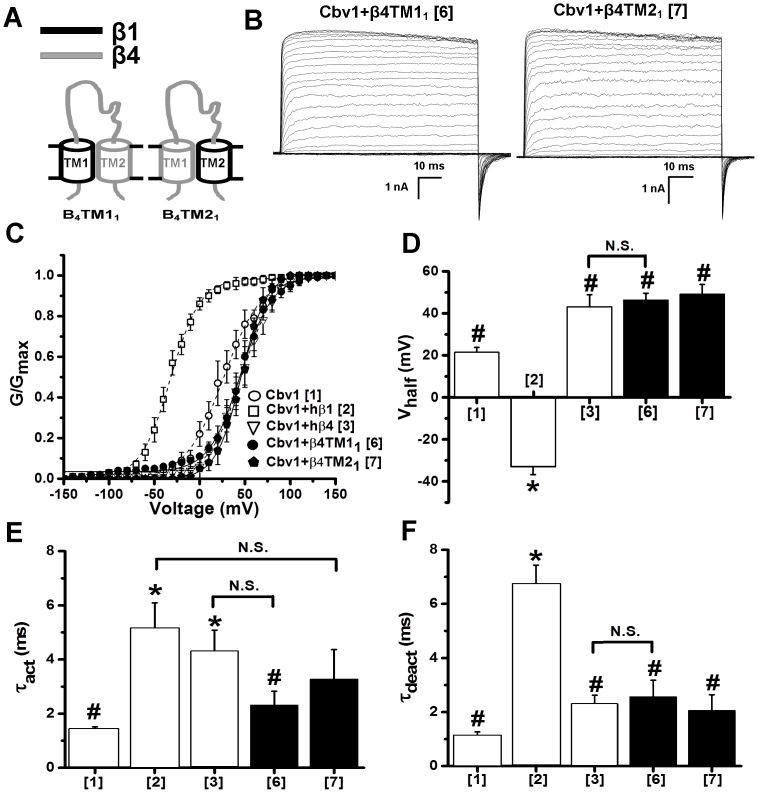
Neither TM1 or TM2 from BK β1 is sufficient to confer the characteristic phenotype of β1-containing to BK channel complexes. (A) Cartoons depicting chimeric constructs that result from swapping individual transmembrane domains (either TM1 or TM2) between hβ1 and hβ4. Regions from β1 and β4 are given in black and grey, respectively. (B) Macroscopic current recordings obtained from I/O oocyte membrane patches expressing cbv1+β4TM1_1_ (construct 6) or cbv1+β4TM2_1_ (construct 7); Ca^2+^_i_ = 10 µM. (C) Averaged G/G_max_-V plots of cbv1, cbv1+hβ1, cbv1+hβ4, cbv1+β4TM1_1_ and cbv1+β4TM2_1_; Ca^2+^_i_ = 10 µM. Averaged V_half_ (D), activation (E) and deactivation (F) time constants (τ_act_, τ_deact_, respectively) obtained cbv1, cbv1+hβ1, cbv1+hβ4, cbv1+β4TM1_1_ and cbv1+β4TM2_1_; Ca^2+^_i_ = 10 µM. *Different from cbv1 (P<0.05); ^#^Different from cbv1+β1 (P<0.05). Error bars correspond to SEM; each point represents the average of ≥4 patches.

To identify whether a particular BK β1 TM was sufficient to determine the hβ1-containing BK channel phenotype, we next engineered hβ1/hβ4 chimeras that contained either TM1 (β4TM1_1_) or TM2 (β4TM2_1_) from hβ1 introduced onto a β4 background, and thus co-expressed such constructs with cbv1 channels (**Fig. 4A**). V_half_-Ca^2+^_i_ plots (**Fig. 4C,D** **and S1C**) and τ_deact_ (**Fig. 4F**) from the resulting cbv1+chimeric β1/β4 heteromers drastically differed from those of cbv1+hβ1. Likewise, τ_act_ from β4TM1_1_ was significantly different from that of cbv1+hβ1, with β4TM2_1_ τ_act_ reaching intermediate values (**Fig. 4E**). Therefore, in contrast to β4TMs_1_ containing both TM segments of β1, β4TM1_1_ and β4TM2_1_ that contained only one of the TM of β1 failed to substitute for hβ1 in characteristically modifying the cbv1 channel phenotype. Therefore, both β1 TMs are required to render the phenotype characteristic of cbv1+hβ1 channels.

**Figure 5:**
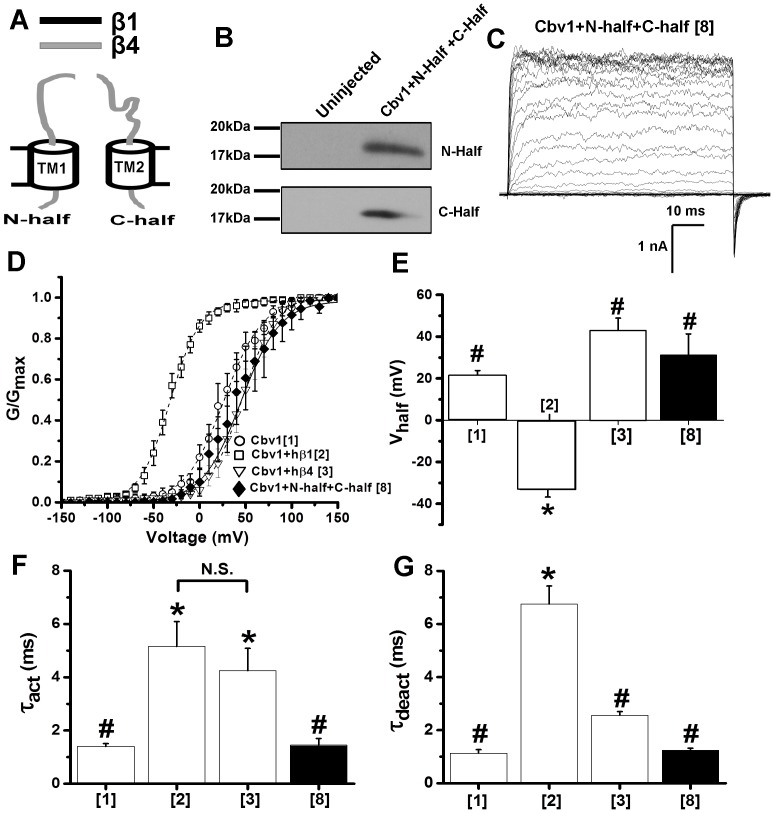
Physical continuity of the EC loop between TM1 and TM2 is essential to confer the characteristic phenotype of β1-containing BK channel complexes. (A) Cartoons depicting two hβ1/hβ4 chimeric constructs termed “N-half” and “C-half”, obtained by cleaving the EC loop between TM1 and TM2 in the β4TMs_1_ chimera. Regions of β1 and β4 are given in black and grey, respectively. When expressed together (panels C-G and main text), “N-half” and “C-half” chimera have been labeled as construct 8. (B) Western blots reflecting the surface presence of N-half and C-half, when co-expressed with cbv1, obtained by surface biotinylation of *Xenopus* oocytes expressing cbv1+N-half+C-half complexes. Blot image where left and right lanes contain samples from uninjected and N-half+C-half chimera-injected oocytes, respectively. (C) Representative traces of macroscopic current recordings obtained from I/O oocyte membrane patches expressing construct 8; Ca^2+^_i_ = 10 µM. (D) Averaged G/G_max_-V plots from cbv1, cbv1+β1, cbv1+β4, and cbv1+construct 8; Ca^2+^_i_ = 10 µM. Averaged V_half_ (E), activation (F) and deactivation (G) time constants (τ_act_, τ_deact_, respectively) obtained cbv1, cbv1+β1, cbv1+β4, and cbv1+construct 8. *Different from cbv1 (P<0.05); ^#^Different from cbv1+β1 (P<0.05). Error bars show SEM; each point represents the average of ≥4 patches.

Finally, we decided to determine whether integrity in the peptidic connection between β1TM1 and β1TM2 *via* the EC loop was necessary to provide the normal phenotype of cbv1+hβ1 channels. Thus, we engineered two “split” chimeras from the β4TMs_1_ to render: 1) “N-half chimera”, which contained the N-terminus from β4, TM1 from β1 and proximal half of β4 EC loop; 2) “C-half chimera”, which contained the distal half of β4 EC loop, TM2 from β1, and the C-terminus from β4 (**Fig. 5A**). After oocyte co-injection of these two chimeras with cbv1, surface expression of both chimeras was confirmed by surface biotinylation (**Fig. 5B**). Electrophysiology data demonstrate that V_half_-Ca^2+^_i_ plots (**Figs. 5C,D** **and S1D**), τ_act_ (**Fig. 5F**) and τ_deact_ (**Fig. 5G**) from the resulting cbv1+chimeric β1/β4 heteromers are not able to reproduce the cbv1+hβ1 phenotype (Fig. 3) but match those of homomeric cbv1 channels. These data indicate that co-expression of each β1 TM surrounded by its “immediate” β4 background (**Fig. 5A**) is not sufficient to render the characteristic cbv1+hβ1 channel phenotype. Rather, a physical connection between two individual β1TM1 and β1TM2 *via* the EC loop is necessary to ensure such phenotype.

## Discussion

In spite of the significant advances in addressing the role of BK β1 subunits in the different aspects of slo1 channel gating and in cell physiology and pathophysiology, the involvement of specific BK β1 regions in determining the characteristic phenotype of β1-containing BK currents remains unresolved. A previous study has shown that the β1 EC loop regulates intrinsic gating and voltage sensor activation. However, data fall short from demonstrating that the β1 EC loop is sufficient to modulate the apparent Ca^2+^_i_ sensitivity of the channel [24], a channel property that is critical for the role of β1-containing BK channels in cell function. On the other hand, studies from BK channels made of slo1 and chimeric β1/β2 subunits indicated that β C- and N-ends played a significant role in determining the channel phenotype, and raised the hypothesis that β TMs contribute to overall functional coupling between α and β subunits [16]. Indeed, our current study clearly demonstrates that neither β1 TM is sufficient but both are necessary to increase the channel's apparent Ca^2+^_i_ sensitivity.

The current data also demonstrate that for both TMs to provide the basic phenotype of beta1-containing BK channels, these two segments must be physically connected, in this case *via* the EC loop of β4 subunit. Noteworthy, EC loops from β1 and β4 share two critical domains that determine V_half_ [24; see next paragraph]. At least two interpretations on this crucial role of an EC loop are possible: 1) the connection between the two TMs *via* the EC loop helps to properly orient both TMs, so each efficiently interacts with a corresponding slo1 functional domain partner. It is interesting to note that disulfide cross-linking assays placed the outer face of β1 TM1 in close proximity to the outer faces of slo1 S1 and S2 domains while placing the outer face of β1 TM2 in the vicinity of the outer face of S0 in the adjacent slo1 subunit [5], [22], [33]. If physical associations match functional coupling (yet restrictions are considered below), the EC loop physical's integrity would allow optimal β1 TM1-slo1 S1/S2 and β1 TM2-slo1 S0 functional coupling; 2) functional coupling between a single β1 TM and its corresponding functional domain in slo1 is translated into modification of phenotype only if such functional coupling imparts a change in conformation/topology of the other β1 TM, such communication between TMs requiring the physical integrity of the EC loop.

Previous studies on the role of EC loop of β1 on BK/β1 channel modulation revealed two critical domains (A and B) in the EC loop that were important to modulate various functional parameters, such as V_half_, τ_act_, τ_deact_ and voltage sensitivity of β1-containing BK channels [24]. Noteworthy, we engineered split chimeras so the N-half chimera contained the ‘A’ domain in its entirety whereas the C-half chimera contained the ‘B’ domain in its entirety (**Fig. 5A**). Our biotinylation data demonstrate that both the N-half and the C-half chimera were properly expressed in the cell membrane. However, neither chimera was sufficient to bring the phenotype of β1-containing BK channels. Moreover, when both half-chimeras were coexpressed with cbv1, they failed to evoke the normal phenotype of β1-containing BK channels. This failure can be explained by some non mutually exclusive possibilities: when physically separated from each other, the half-chimeras, while present in the membrane, failed to acquire the proper stoichiometry and/or conformational association with the channel-forming subunits [see above). In synthesis, our results indicate that segments A and B *per se* are not sufficient to provide the normal phenotype of β1-containing BK channels but a physical connection between the two β1 halves is needed.

Remarkably, both BK beta1 and beta2 increase the channel's apparent calcium sensitivity to a similar degree [1], [17]. We speculate that this similar change in phenotype recognizes a similar physical association between these regulatory subunits and slo1. Using a TOX-CAT assay, Morera et al. (2012) [34] have demonstrated a physical association between β2 TM1 and slo1 S1 whereas physical interactions between slo1 and other β regions (EC loop, TM2) could not be observed. Our functional data, however, show that the β1 N-half chimera, which includes TM1, while fully expressed in the membrane (**Fig. 5B**), failed to alter slo1 current phenotype. Thus, as previously documented by cys-substitutions that alter slo1-β subunit physical association but not phenotype [35], physical association is not sufficient to document functional coupling between β and α BK subunits.

Our current data provide critical information over previous findings on the structural bases of BK channel function regulation by accessory subunits. Using chimeric channels made by swapping mslo1/hslo1 and dslo regions it has been shown that the slo1 N-end and S0 are both critical for channel function regulation by β1 [4], [21], [35]. In addition, disulfide cross-linking studies have shown that the N-terminal EC end of slo1 S0 is in close proximity to its S3 and S4 segments. These three segments (S0, S3, S4) are thought to move in concert during voltage sensor activation [5], [20]. Consequently, substitutions in S0 disrupt the voltage-dependent activation of BK channels, underscoring the critical role of S0 in channel function [36]. Disulfide cross-linking studies [5], [20] show that BK β TM1 and TM2 are both packed close to each other at the mouth of the cleft between the voltage sensing domain (VSD) of two adjacent slo1 [5], [20]. Within this cleft, TM1 is close to S1 of one VSD and TM2 close to S0 of the adjacent VSD [5], [20], [22]. The proposed location of both TM1 and TM2 and our current data raise the hypothesis that β1 TMs and slo1 VSD are functionally coupled within membrane-spanning regions themselves, the VSD serving as a scaffold for proper β1 subunit conformation.

In conclusion, from a combination of patch-clamp electrophysiology on BK channels made of cbv1 and native or chimeric beta subunits, and surface biotinylation, we demonstrate for the first time that both transmembrane domains of BK β1 are required to provide the characteristic ion current phenotype of beta1-containing BK channels. Moreover, BK β1 transmembrane regions need to be physically linked by the EC loop in order to control essential parameters of BK current, such as V_half_, activation and deactivation kinetics. Current information will lead to pinpoint mutagenesis strategies to identify the specific amino acid residues that are involved in providing the phenotype of β1-containing BK channels, which represents a first necessary step to understand how β1 couples to channel-forming slo1 proteins and to design selective BK β1-targeting agents and modify tissue physiology in a rather selective manner.

## Supporting Information

Figure S1 **Both TMs of BK β1 subunit are required for conferring the characteristic phenotype (V_half_) of β1-containing BK channel complexes.** Averaged V_half_-Ca^2+^_i_ plots for constructs 1-8, obtained over a wide range of Ca^2+^_i_ levels (nominal zero to 100 µM). (A) β1-subunit (construct 2) causes an increase in the channel's apparent Ca^2+^ sensitivity (which is more evident at>1 µM Ca^2+^_i_), whereas β4 (construct 3) does not. (B) β1/β4 chimera containing both TM domains from β1 (construct 5) reproduces the characteristic phenotype (e.g, V_half_) of β1-containing BK channels over a wide range of Ca^2+^_i_, including physiological levels found nearby the BK channel during smooth muscle contraction. (C) Chimeras containing individual TM domains (TM1 and TM2) from β1 (constructs 6 and 7) fail to mimic the V_half_-Ca^2+^_i_ relationship of β1-containing BK channels. (D) Cleaving the EC loop between TM1 and TM2 in the β4TMs1 chimera (construct 8) also fails to reproduce the normal V_half_-Ca^2+^_i_ relationship of BK β1 channels. Error bars correspond to SEM; each point represents the average of ≥4 patches.
(TIF)
