# Supplementary figures and images for: Both Transmembrane Domains of BK β1 Subunits Are Essential to Confer the Normal Phenotype of β1-Containing BK Channels
Source: PLoS One. 2014 Oct 2;9(10):e109306. doi: 10.1371/journal.pone.0109306 (PMC4183656; doi:10.1371/journal.pone.0109306)

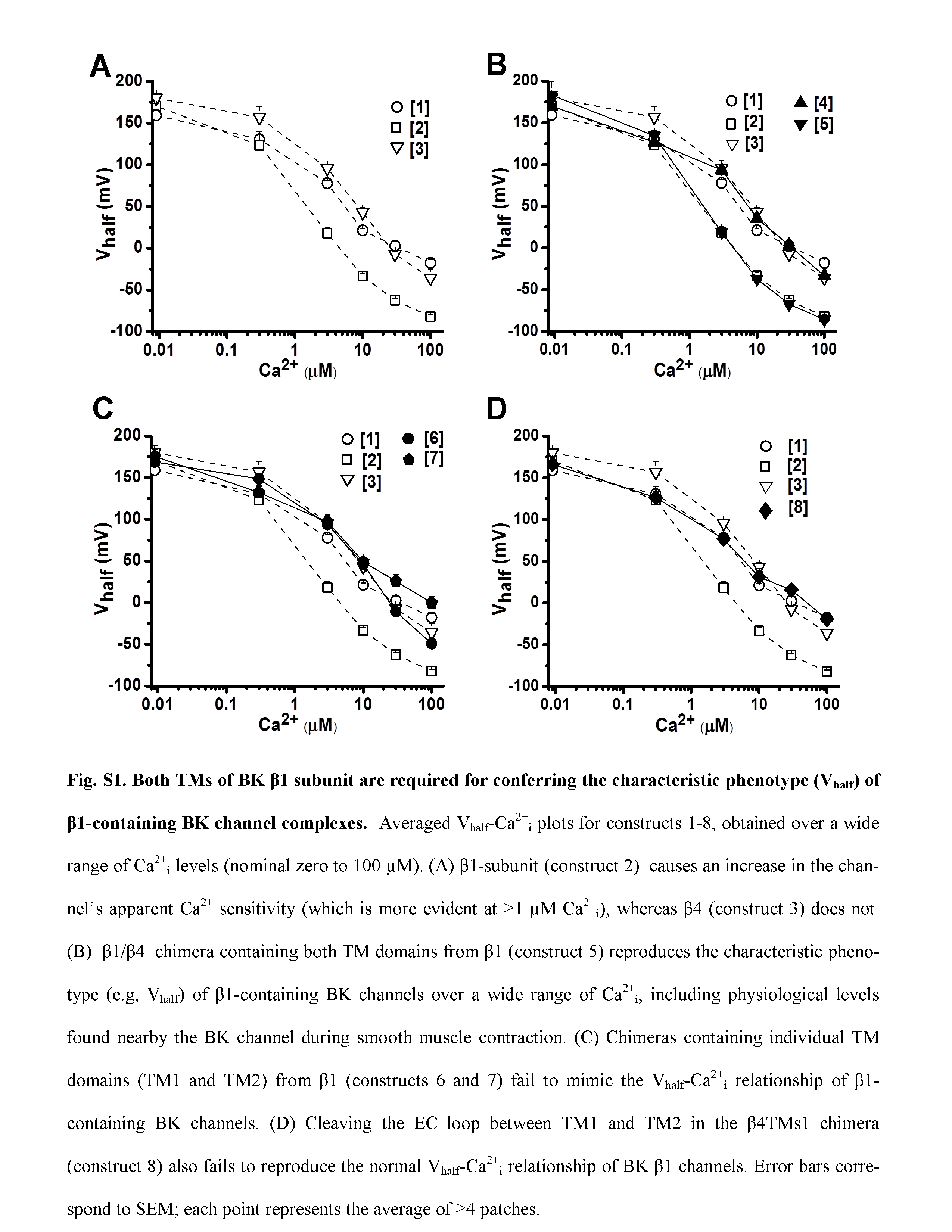

Supplement: Figure S1 — Both TMs of BK β1 subunit are required for conferring the characteristic phenotype (Vhalf) of β1-containing BK channel complexes. Averaged Vhalf-Ca2+ i plots for constructs 1-8, obtained over a wide range of Ca2+ i levels (nominal zero to 100 µM). (A) β1-subunit (construct 2) causes an increase in the channel's apparent Ca2+ sensitivity (which is more evident at>1 µM Ca2+ i), whereas β4 (construct 3) does not. (B) β1/β4 chimera containing both TM domains from β1 (construct 5) reproduces the characteristic phenotype (e.g, Vhalf) of β1-containing BK channels over a wide range of Ca2+ i, including physiological levels found nearby the BK channel during smooth muscle contraction. (C) Chimeras containing individual TM domains (TM1 and TM2) from β1 (constructs 6 and 7) fail to mimic the Vhalf-Ca2+ i relationship of β1-containing BK channels. (D) Cleaving the EC loop between TM1 and TM2 in the β4TMs1 chimera (construct 8) also fails to reproduce the normal Vhalf-Ca2+ i relationship of BK β1 channels. Error bars correspond to SEM; each point represents the average of ≥4 patches. (TIF) [file pone.0109306.s001.tif]
